# Assessment of Residual Chlorine Interaction with Different Microelements in Stormwater Sediments

**DOI:** 10.3390/molecules28145358

**Published:** 2023-07-12

**Authors:** Marina Valentukeviciene, Ieva Andriulaityte, Viktoras Chadysas

**Affiliations:** 1 Department of Environmental Protection and Water Engineering, Faculty of Environment Engineering, Vilnius Gediminas Technical University, LT-10223 Vilnius, Lithuania; ieva.andriulaityte@vilniustech.lt; 2 Department of Mathematical Statistics, Faculty of Fundamentals Science, Vilnius Gediminas Technical University, LT-10223 Vilnius, Lithuania

**Keywords:** stormwater, disinfection processes, chlorine, data analysis

## Abstract

One consequence of intensive outdoor disinfection using chlorinated compounds is environmental pollution. It has been found that disinfectants are the most effective tool to avoid the spread of infections and viruses. Studies have shown that the use of chlorine-based disinfectants (sodium hypochlorite) leaves residual chlorine and other disinfection byproducts in the environment. They also have harmful effects on, inter alia, water quality, ecosystems, as well as exacerbating the corrosion of surfaces. To meet regulatory standards, monitoring of the presence of residual chlorine in the environment is vitally important. The aim of this study is to analyse the occurrence of residual chlorine in stormwater after outdoor disinfection using sodium hypochlorite and to investigate its interaction with different microelements as well their possible impacts. Stormwater samples collected at permanently disinfected locations were analysed via X-ray absorption spectroscopy. The concentrations of Cl and the following elements Na, Si, K, Ca, Cr, Fe, Ni, Cu, Zn were detected and their relationship with chlorine was determined using the Python programming language. The research presents Cl concentration values (%) that vary from 0.02 to 0.04. The results of the modelling revealed strong correlations between Cl and Fe (value 0.65) and Ca (value −0.61) and the occurrence of CaCl_2_ and FeCl_3_. The strong relationship between Cl and Fe explains the significant increase in surface corrosion after disinfection with chlorine-based substances.

## 1. Introduction

In many countries, surface water is a source of drinking water. It follows that stormwater treatment must meet the highest standards in order to negate the impact of harmful substances on ecosystems such as rivers and lakes. Research categorises contaminants present in stormwater as follows: heavy metals; pollutant organic compounds; and other pollutants [1]. Such pollutants entering bodies of water due to runoff harm the environment destroys the natural water ecosystems [2,3]. Furthermore, high water turbidity also affects water quality [4,5], etc.

In recent years, there has been a marked increase in environmental pollution caused by chlorinated compounds used in intense disinfection processes. Chlorine-based disinfectants are widely applied to clean outdoor and indoor spaces to deactivate various viruses and microorganisms. Researchers have assumed that increased use of disinfectants is the primary cause of elevated levels of disinfection by-products and residual chlorine in the environment, as well as their negative effects on ecosystems [6,7,8]. Following disinfection, chlorinated compounds and residual chlorine enter stormwater and surface water bodies where residual chlorine reacts with water to form chemical compounds such as hypochlorous acid, chloride ion, and hypochlorite. Current research shows that high doses of residual chlorine lead to the formation of harmful disinfectant by-products, which can pose toxic effects to organisms and increase the risk to the environment and health [9,10,11], corrode surfaces, reduce water quality, etc. With this in mind, we need to monitor, accurately and in a timely manner, the amount of residual chlorine in the environment to ensure the set regulatory level is maintained [12]. Therefore, there is an increasing need for further research.

Disinfection with sodium hypochlorite is carried out mainly in public spaces. This chemical compound is characterised by high energy consumption, strong corrosiveness, high pollution, and high toxicity. It is also a stable chemical, easy to apply and control, and has low operating and preparation costs [13,14]. Sodium hypochlorite in aqueous solutions dissociates into residual chlorine. Studies show that it is an effective means of fighting viruses [15]. Disinfection by sodium hypochlorite is described as NaOCl + H_2_O → Na^+^ + HOCl + OH^−^ (HOCl → H^+^ + OCl^−^) [16]. Sodium hypochlorite releases free chlorine in water environment which reacts with water and from hypochlorous and hydrochloric acids as a result. The reaction is described as the following: Cl_2_ + H_2_O → HOCl + HCl. Later, depending on stormwater pH, hypochlorus acid may be released into hypochlorite (OCl^−^) and hydrogen (H^+^) ions (HOCl → H^+^ + OCl^−^). The World Health Organization recommends applying sodium hypochlorite at 0.1% (1000 ppm) for outdoor and indoor surface disinfection [17]. According to European Union regulations [18], sodium hypochlorite can be used for outdoor surface decontamination. The hypochlorite ion is in equilibrium with hypochlorous acid and chlorine, depending on the pH level. When the pH is below 4, chlorine is available, whereas for hypochlorous acid, the pH should be neutral. The hypochlorite ion is available when the pH value is greater than 10. Studies show that low concentrations of residual chlorine can impact bacteria species in water [19,20]. Residual chlorine can react with dissolved organic matter, forming chlorinated disinfection by-products (e.g., chloroform), many of which are biologically toxic. Residual chlorine interacts with the majority of metals and non-metals, such as alkaline and alkaline earth metals. Chlorine has a higher oxidation potential than chlorine compounds, including chloramine, dichloramine, nitrogen trichloride, and other organic compounds [21]. Recent research reported on the relationship between disinfectant concentrations (sodium hypochlorite) and stormwater quality. Fluctuations in chlorine-based disinfectant concentrations have an immediate impact on stormwater pollution indicators, e.g., pH, conductivity, turbidity and colour [22], and also have an impact on aquatic microorganisms [23]. Studies have reported that the interaction of residual chlorine containing chemical substances (disinfectants) with organic and inorganic pollutants in water leads to the formation of disinfectant by-products: haloacetic acids, trihalomethanes, dibromochloromethane, bromodichloromethane, tribromethane, tribromoethane, dichloroacetonitrile, chloramines and other organic compounds [24,25]. The year 2022, generally agreed to be the last year of the pandemic, showed the need to strengthen stormwater quality monitoring to reduce residual chlorine concentrations in water bodies. This calls for strong and global cooperation between academia, industry, and national governments. However, science shows that despite the environmental contamination resulting from the disinfectant process, there is a lack of data on the extent of its impact (considered to be enormous) and what methods could be applied to evaluate the effect of disinfection by sodium hypochlorite and other chemical substances [7,26]. More analysis and research are needed to determine the long-term effects on aquatic ecosystems and possible treatment technologies.

The novelty of this research is that the interactions of residual chlorine in the environment have not been widely investigated. The objective of the article is to analyse the occurrence of residual chlorine in stormwater after disinfection and its interaction with different elements.

## 2. Results and Discussions

Research on the impact of chlorine-based disinfectants is usually related to drinking water and wastewater plants [27,28]. However, this study was designed to analyse how surface disinfection causes stormwater pollution, including residual chlorine migration into the environment. However, outdoor spaces at nursing homes, wellness and SPA centers, restaurants and other sensitive areas that experience very high levels of foot traffic should be disinfected regularly to avoid viruses or infections. At the time of writing, the probability of new and existing pandemics is increasing, so that outdoor space disinfection may become a daily process in many countries in the near future. Therefore, there is a pressing need to assess the long-term impact of disinfectants on the environment. This research is ongoing. Initial data on the impact of chlorine-based disinfectants on stormwater pollution indicators [22] show that by increasing the amount of disinfectants, the stormwater pollution indicators change and residual chlorine concentrations are detected. This study is focused on the relationship of evaluation of the sodium hypochlorite with various microelements and its possible interaction and dependencies.

**Figure 1:**
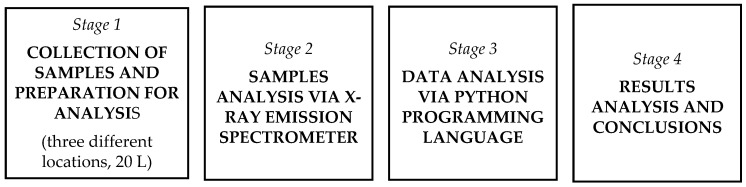
Principle scheme of experiments.

The quality of stormwater collected from non-disinfected areas was constantly measured using standard methods and compared with stormwater samples obtained from permanently disinfected places. This will explain various negative phenomena in the environment. Previous research has revealed that disinfection of outdoor spaces with chlorine-based substances (sodium hypochlorite) creates the conditions for surface corrosion and contributes to deterioration in environment conditions, etc. Accordingly, the chlorine reactivity Cl is defined as a strong oxidiser and a highly active element that reacts with almost all metals and nonmetals. Scientific sources note that chlorine compounds form stable compounds more easily with alkali earth elements such as Na, K, Ca, and Mg [21]. X-ray sample analyses (standart deviation 0.00012–0.04) have detected concentrations of Cl and the following microelements Na, Si, K, Ca, Cr, Fe, Ni, Cu, and Zn after disinfection, and date analysis using Python reported strong Cl correlations with several elements (Fe, Ca). The principle scheme of experiments and detailed description is presented in Figure 1 and in Section 3.

### 2.1. Data Analysis

Data analysis was based on the evaluation of information obtained from test water samples collected at different locations within the largest region of Lithuania. Water samples for analysis were taken at stormwater outlets in nursing home areas (disinfected and nondisinfected places). During the pandemic, the latter were permanently disinfected with sodium hypochlorite to suppress virus transmission.

**Figure 2:**
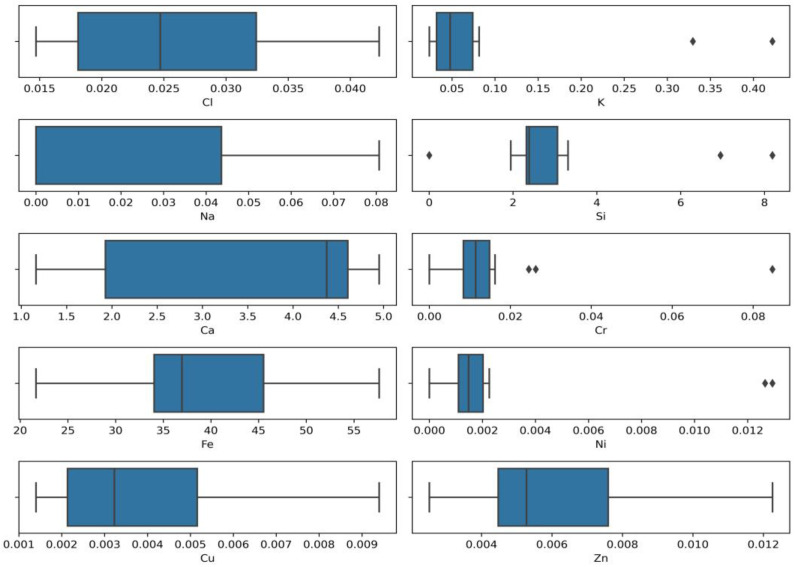
Box plots of elements distribution (concentrations, %).

Outdoor disinfection was carried out using sodium hypochlorite following the World Health Organization recommendations (0.1% (1000 ppm)) [17]. Studies have revealed that surface decontamination using chlorine containing disinfectants leads to the release of residual chlorine. Residual chlorine is formed by sodium hypochlorite ions and may also exist as combined chlorine (inorganic and organic chloramines) [16]. The research also focused on the detection of residual chlorine and the amount of other elements and their mutual interaction. The results show that the characteristics (pH, conductivity, turbidity, colour) of the collected stormwater samples (disinfected and non-disinfected areas) are almost the same, and residual chlorine migration in the environment depends on surface material (metal, concrete, soil) and area. Python programming language libraries were used to present the values of the initial data distribution and outliers of detected elements. The results are given the Box plots (Figure 2).

Figure 2 reveals the clear outlier of potassium (K), silicon (Si), chromium (Cr), nickel (Ni), and also shows that the values scales of these four elements differ quite significantly. The analysis using the Python chlorine (Cl) box plot shows a normal distribution with iron (Fe) and copper (Cu) distributions positively skewed. In contrast, the calcium (Ca) and sodium (Na) distribution is negatively skewed. It is assumed that different values of detected elements in test samples and outliers of elements might be caused by the different sampling locations, the time when sampling was performed, and other conditions such as interruptions caused by routine maintenance and repair work.

**Figure 3:**
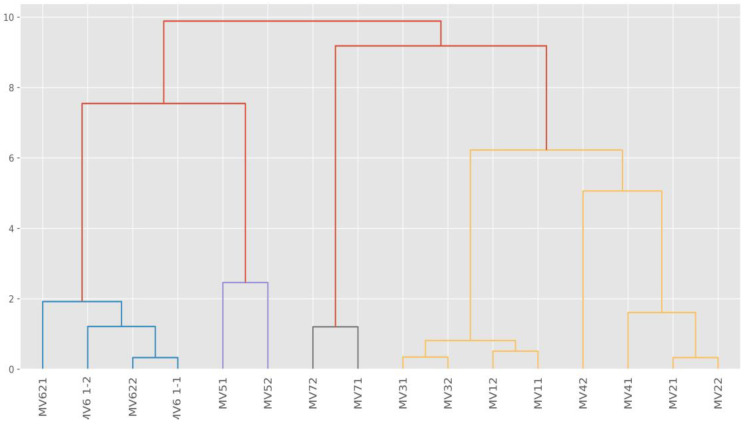
Sample dendogram of elements groups hierarchical clustering (MV—measured values).

The hierarchical clustering method was used to explain possible chlorine interactions with detected elements and their reactions and possible negative impacts on the environment. The results of the sample clustering are presented in Table 1 and Figure 3.

Hierarchical clustering is used to explain similarities of groups of elements and is based on the idea that close objects are more similar to each other than distant objects. The samples were clustered on the Euclidean distance measurement. Table 1 shows that C and Cluster 5 are the closest. The averages of the Na, K, Ni, Cu, and the Zn amounts are the same, and Si, Cl, Ca, Cr, and Fe values are quite similar. The highest values of Cl (0.04%) and Fe (54.97%) concentrations were detected in Cluster 4. One explanation for this is that the old stainless steel provokes heavy corrosion because of intensive disinfection by chlorine-based biocides. Cl is a halogen, and Fe is a transition metal according to the Periodic Table of Elements. Both elements are likely present in the oxidation and reduction processes and produce various compounds such as FeCl_3_, CaCl_2_. Table 1 reveals that the Cu value (0.01%) was obtained with chlorine values of 0.02% (group 2) and 0.04% (Cluster 4). It is assumed that where Cu occurs at heavy metal locations in stormwater outlets, the principal immobilisation mechanism by chlorine compounds is the ion exchange process.

Graphically, hierarchical clustering is expressed by the sample dendogram (Figure 3) which shows similarities and differences between clusters.

Figure 3 shows the differing impact of disinfection. Based on the obtained Cl values in Cluster 1 and Cluster 5, we can conclude that impact chlorination was low and no significant corrosiveness was detected. Cluster 4 presents the highest Cl and Fe values, which explains the high level of surface corrosion at sampling locations. Cluster 3 indicates very high Si concentration (7.57%) (Table 1). This might be explained by the occurrence of chlorine on the surface of the sand particles.

### 2.2. Correlation Analysis

**Figure 4:**
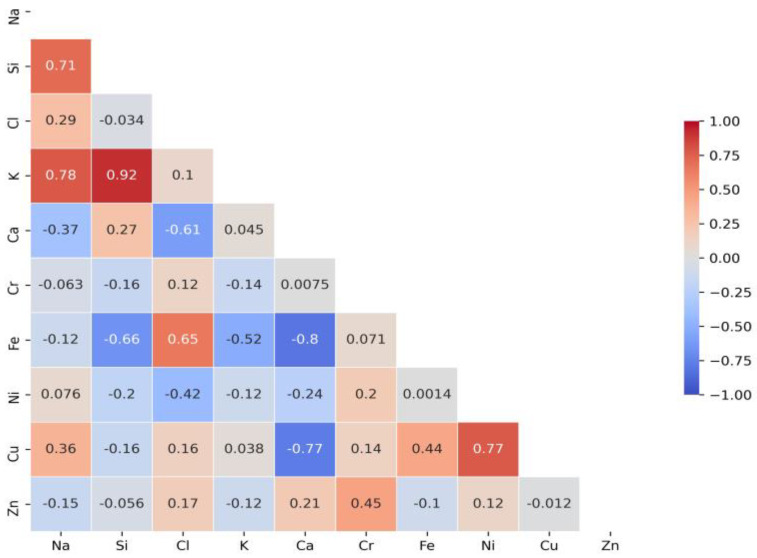
A correlation matrix of elements in test samples.

A correlation matrix is displayed to determine the relationship between different elements. It was applied to summarise research data and as a diagnostic for advanced analyses. Each cell in the table shows the correlation between two variables. Figure 4 presents significant Cl correlations with elements detected via X-ray analysis in test water samples collected after disinfection with chlorine-based biocides at stormwater outlets.

In order to determine which of the established relationships are significant, a further test raised the hypothesis (null hypothesis: H_0_: *ρ* = 0; alternative hypothesis: H: *ρ* ≠ 0). The significant intercorrelation coefficients are given in Table 2. Intercorrelation is significant when values are above 0.5.

Table 2 shows a strong relationship with Fe and Ca. The intercorrelation values are 0.65 (Fe) and −0.61 (Ca). Following the obtained results of significant dependencies between analysed elements, it is assumed that low-solubility compounds of Cl are formed by complexing inorganic ligands (chlorides, etc.). Significant Cl dependencies with Fe and Ca might cause the formation of the following compounds FeCl_3_ and CaCl_2_. The residual chlorine impact on the formation of iron ions and reveal linear relationship between concentrations of iron and the residual chlorine [29]. The strong relationship of Cl with Fe explains the surface corrosion that occurs after disinfection with chlorine. Data analysis (Table 2) has also shown strong relationship between Si and K (0.92), Na and K (0.78), Ni and Cu (0.77), Ca and Fe (−0.8), Ca and Cu (−0.77), Si and Fe (−0.66). Previous research [15] has demonstrated the presence of silicon concentrations in stormwater after disinfection. It was assumed that Cl was formed on the surface of the sand particles; however, correlation analysis did not determine any linear relationship between Cl and Si. Some research has find out the significant positive correlation among Ca, Mg, Cl. Na, K, and Cl concentrations also correlated strongly [30]. The most efficient element combination models were obtained via the values of the residual sum of squares and the determination coefficient (Table 3).

The most efficient regression models with different numbers of elements were identified based on the RSS and R^2^ values. Table 3 shows that the best combination is formed by these elements K, Fe, Cu, Zn.

Standardised values of these elements are used for the regression model that aims to predict Cl values. A Python programming language is used to evaluate the coefficients of the model. The following model was obtained on the results of correlation.

C_Cl_ = 0.8779 C_K_ + 1.3531 C_Fe_ < 0.6628 C_Cu_ + 0.4155 C_Zn_ (1)

**Figure 5:**
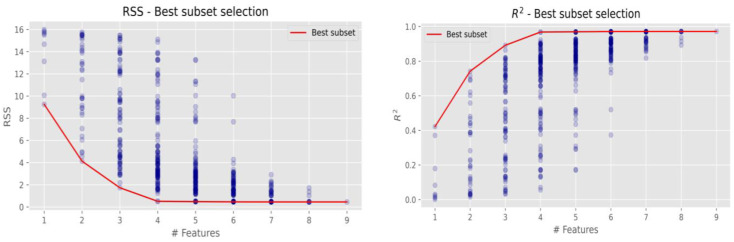
The most efficient regression models of elements groups.

Research data demonstrate that Cl concentration occurred in the environment more than twice that of K and four times higher of that on Zn. When the concentrations of the above-mentioned elements increase, the concentrations of Cl also increase. Table 3 shows that Cu has an inverse impact on Cl: when Cu concentration is higher, Cl concentration decreases. To determine the most efficient regression model among them, it is necessary to compare the trends of efficiency change when an increasing number of elements are included. The best models are given in Figure 5.

Mostly, it is better to take a simpler model with fewer variables than a more complex model with more variables, because the decrease in residual sum of squares (RSS) or the increase in determination coefficient (R^2^) is not significant. The lower sample size could ensure the more variation of efficiency indicators. Therefore, a combination of variables with a slightly higher RSS or a lower R^2^ value can be a better option. Figure 5 presents graphically that the best Cl combination via RSS and R^2^ is with four elements (K, Fe, Cu, and Zn); this combination was the lowest with RSS and the highest with R^2^ out of all combinations of each of the four elements. The grouping of more elements and further analysis of other possible element combinations revealed similar results (red lines in both figures), and no significant combinations were detected. It is assumed that the metallic surfaces were affected by the oxidation state of the following elements K, Fe, Cu, and Zn. Corrosive dry deposition can affect building materials when iron corrodes and forms rust and changes the composition of metals by exposure to stormwater or a disinfectant solution (residual sodium hypochlorite). Research reported that sodium hypochlorite or other chlorine-containing disinfectants impact corrosion of the steel after disinfection [31]. Similar results obtained research [30,32,33]; the sample immersed in 5% NaClO for three weeks presented corrosion, a phenomenon that is explained by the beginning of the corrosion in the materials. Water quality analysis were selected: pH, turbidity, residual chlorine, potassium ion, calcium hardness, total hardness, conductivity, total iron, chloride ion, and suspended matter. Accordingly, to the presented research it is necessary to consider various corrosion indices and plan the maintenance procedures following the newest recommendations [33].

## 3. Materials and Methods

### 3.1. Sampling Location and Data Collection

Water samples for data analysis were collected at stormwater outlets in locations that are constantly disinfected—nursing home areas in the Vilnius region, Lithuania.

The main criteria for sampling selection were:

- Location type: areas with the need to apply permanent outdoor disinfection to avoid the spread of virus and infections.
- Geographic characteristics: green area near surface water bodies.

Test samples (20 L) were collected in continuously disinfected areas at stormwater piplines and pipes not connected to disinfected areas. All measured metals in stormwater were obtained from non-disinfected locations and were below detection limits (Na < 0.01%; K < 0.0012%; Ca < 0.004%; Cr < 0.0013%; Fe < 0.03%; Ni < 0.00047%; Cu < 0.00034%; Zn < 0.00022%). Both sampling areas were covered with fine grains of sand to prevent them becoming slippery in winter time, concentrations were detected between 1.66–7.57%. With this in mind, stormwater test samples were taken in three different places in outdoor areas of nursing homes (Vilnius region, Lithuania), all of which are located near surface water bodies. The sampling was carried out according to ISO standards. A one-litre sampling container was washed with distilled water prior to use. For a short time before analysis, test samples obtained at stormwater outlets were placed in hermetically sealed plastic containers and stored at 4 °C. The amounts of chlorine and of the nine following elements Na, Si, K, Ca, Cr, Fe, Ni, Cu, and Zn were analysed via X-ray absortion spectroscopy (emission spectrometer). Metals were determined via the energy dispersive X-ray fluorescence (EDXRF) method. The samples were treated following the procedures of homogenisation using MM 400 (the mixer mill is a compact versatile benchtop unit which has been developed for small dry and wet small amounts of the sample), homogenisation with Licowax (4 g of sample and 0.9 g of polyethylene wax), and pressing with a PP15 press into 32 mm pellets (two for every sample). The primary content of the investigated metals was determined by EDXRF by using SPECTRO XEPOS equipment (Germany) and the TURBOQUANT calibration method for pressed pellets. The numbers obtained were then recalibrated by using 44 ISE certified reference materials, CRM 2709 and CRM 2711 and all concentrations in collected samples were reported as weight per cent (wt%) contents. The concentrations of these nine elements were determined in each test sample. The elements evaluated were within the acceptance criteria of the method of 10% of the known value, and the deviation for most elements was less than 3%.

### 3.2. Data Analysis Methodology

Data analysis was carried out using the Python programming language. Python was selected because of the adaptable and efficient nature of its programming features. Scientists recommend utilising Python for data analysis due to its ability to work with a variety of modules, and also because it offers more options in graphic packages and data visualisation [34,35]. Furthermore, the use of Python in data science continues to grow because it can be applied to a wide number of libraries to model research results [36,37,38]. To describe the interactions of microelements, predict their possible effects, and express and explain the obtained results visually and graphically, this study used the following libraries: Pandas for data preparation, Matplotlib and Seaborn for data visualisation, Scipy for statistical hypothesis testing, and Scikit-learn for clustering and building regression models.

The research investigated the amount of chlorine (Cl) present in stormwater after disinfection, and determined the impact of other elements on Cl to analyse whether chlorine released into the environment reacts with various elements and causes surface corrosion and other negative factors. The research showed that during disinfection, organic substances decompose into simple substances that might be more easily removed by natural reactions.

Storm water samples were collected at different locations; therefore, the values differ significantly. Hierarchical clustering, an algorithm applied to group similar data into groups (*clusters*), was used to confirm and explain this occurrence. The end point is usually a set of clusters in which the clusters differ from each other. The objects within each cluster are also similar to each other. As the scales of the values of the elements are different, in order to prevent one element from influencing the results of the analysis, the initial data were standardised using the following formula:

(2) $$z_{i}=\frac{x_{i-\mu }}{\sigma }$$

where *µ*—mean of the values; σ—standard deviation.

Correlation analysis was used to assess the statistical relationship between elements. The relationship between the two variables was determined on the basis of the Pearson correlation coefficient.

(3) $$r_{xy}=\frac{{n\sum x}_{i}y_{i}-\sum x_{i}\sum y_{i}}{\sqrt{n\sum x_{i}^{2}-{\left(x_{i}\right)}^{2}}\sqrt{n\sum y_{i}^{2}-{\left(y_{i}\right)}^{2}}}$$

where *n* is the number of observations, $x_{i}$—value of *x* (for *i*-th observation), $y_{i}$—value of *y* (for *i*-th observation).

The estimated correlation coefficient reflects only the sample and not the population as a whole. Explaining common statistical concepts in experimental research is necessary to ensure scientific rigour, clarity, and appropriate interpretation of results. The Pearson correlation coefficient and the definition of statistical significance in experimental research are important for the following reasons: This information helps to understand why this particular statistical analysis was used and provides more clear information on the interpretation of the results; it provides also an opportunity for other researchers to replicate the research and to base further studies on the results obtained, and ensures that the research’s conclusions are appropriate and scientifically based. Finally, it confirms that the research was transparent and reliable. Furthermore, a hypothesis test of significance of the correlation coefficient was carried out to determine whether the linear relationship between the sample data are strong enough to be confirmed. The hypothesis test allowed us to determine whether the value of the population correlation coefficient *ρ* is close to zero or significantly different from zero. This was decided on the basis of the sample correlation coefficient r and the sample size n. To make sure that the correlation coefficient was statistically significant, the following hypothesis was tested.

The null hypothesis assumes that there is no relationship between the variables, while the alternative hypothesis suggests that there is a correlation between them. The *p*-value method is used in hypothesis testing to check the significance of the given null hypothesis. Then, deciding to reject or support it is based on the specified significance level. If the *p*-value is less than a predetermined significance level (typically 0.05), the null hypothesis is rejected, and the alternative hypothesis is accepted, indicating the presence of a correlation between the variables.

If the *p*-value is not less than the statistical significance (α = 0.05), the null hypothesis is accepted. This means that the correlation coefficient does not differ significantly from 0 and the dependance is not significant. Otherwise, if the *p*-value is less than the significance level (α = 0.05), the null hypothesis is rejected. This means that there is sufficient evidence to conclude that there is a significant linear relationship between elements because the correlation coefficient is significantly different from zero. The Python package, scipy.stats, was used to calculate the *p*-value.

Regression models were constructed to determine the statistical relationship between Cl and the other elements. The models were built using different combinations of elements:

- All possible models by adding single elements;
- All possible models by adding couple elements;
- All possible models by adding all combinations until all elements are included in the model.

Since the model can include many different elements in total, 2^9^ = 512 different models were developed. The efficiency was determined through reference to the residual sum of squares (RSS), or by the highest value of the determination coefficient R^2^. RSS helps to identify the level in errors of the regression model. Lower RSS shows the better model‘s fit to the data. The determination coefficient R^2^ is used to determine the part of variance of the dependent variable that can be explained by the independent variable. R^2^ presents how the data fit the regression model. The higher value of R^2^ means that the model fits the data well.

## 4. Conclusions

Utilising the Python programming language revealed significant outliers of potassium (K), silicon (Si), chromium (Cr), and nickel (Ni). Data analysis established the normal distribution of chlorine (Cl), positively skewed distribution of iron (Fe) and copper (Cu), and negatively skewed distribution of calcium (Ca) and sodium (Na).

Research defined Cl strong relationship with Fe and Ca. The inter-correlation values were 0.65 (Fe) and −0.61 (Ca). It is assumed that significant Cl dependencies with Fe and Ca cause the formation of the following compounds: FeCl_3_; and CaCl_2_. The strong relationship of Cl with Fe explains surface corrosion after disinfection with chlorine-based substances.

Regression analysis of Cl showed that the most significant combination is formed by K, Fe, Cu, and Zn. Cl occurred in the environment after sodium hypochlorite and is the most affected by Fe; the impact of Fe on Cl is twice that on K and four times higher that on Zn. If the concentrations of the above-mentioned elements increase, the amount of Cl also increases.

Research has detected the concentrations of residual chlorine and Na, Si, K, Ca, Cr, Fe, Ni, Cu, and Zn after disinfection. It has revealed that outdoor disinfection using chlorine-based disinfectants (sodium hypochlorite) causes surface corrosion and contributes to the deterioration of environmental conditions. Chlorine is a strong oxidiser and a very active element that reacts with almost all metals and non-metals.

More research is needed on the impact on the environment and different surfaces of chlorine-based disinfectants. Studies shows that more parameters recommended for the investigation in the future following newly provided research [39,40,41].

## Figures and Tables

**Table 1:** Averages of element amounts (%) in the identified clusters.

Cluster	Na	Si	Cl	K	Ca	Cr	Fe	Ni	Cu	Zn
1	0.00	2.70	0.03	0.04	4.71	0.03	35.56	0.00	0.00	0.01
2	0.03	1.66	0.02	0.03	2.09	0.03	43.09	0.01	0.01	0.01
3	0.08	7.57	0.03	0.38	4.03	0.01	22.12	0.00	0.00	0.01
4	0.03	2.24	0.04	0.07	1.32	0.01	54.97	0.00	0.01	0.01
5	0.00	2.53	0.02	0.04	4.63	0.01	35.27	0.00	0.00	0.00

**Table 2:** Significant dependencies of elements.

Pair of Materials	*ρ*	*p*-Values
Si, K	0.92	0.0000
Ca, Fe	−0.80	0.0002
Na, K	0.78	0.0004
Ni, Ci	0.77	0.0005
Ca, Cu	−0.77	0.0004
Na, Si	0.71	0.0022
Si, Fe	−0.66	0.0056
Cl, Fe	0.65	0.0065
Cl, Ca	−0.61	0.0123
K, Fe	−0.52	0.0375

**Table 3:** Efficient regression models of elements groups.

Variables	RSS	R^2^	Variables
1	9.253943	0.421629	(Fe)
2	4.127872	0.742008	(Ni, Cu)
3	1.732323	0.891730	(Ca, Ni, Zn)
4	0.507963	0.968252	(K, Fe, Cu, Zn)
5	0.481758	0.969890	(K, Fe, Ni, Cu, Zn)
6	0.458953	0.971315	(K, Cr, Fe, Ni, Cu, Zn)
7	0.454023	0.971624	(K, Ca, Cr, Fe, Ni, Cu, Zn)
8	0.453742	0.971641	(Na, K, Ca, Cr, Fe, Ni, Cu, Zn)
All	0.453187	0.971676	(Na, Si, K, Ca, Cr, Fe, Ni, Cu, Zn)

## Data Availability

The data presented in this study are available on request from the corresponding author.

## Sample Availability

Samples of the compounds are available from the authors upon request.
